# Dr. Balfour on the Vienna School and on Homœopathy

**Published:** 1847-04

**Authors:** George W. Balfour


### V. REMARKS ON THE DISEASES OF VIENNA, AND ON THE COMPARATIVE EFFECTS OF THE HOMŒOPATHIC, HEROIC, AND EXPECTANT OR SIMPLE METHODS OF TREATMENT.

BY GEORGE W. BALFOUR, M.D.

(*In a Letter to John Forbes, M.D., F.R.S.*)

Corstorphine, March 13, 1847.

MY DEAR SIR,—You will oblige me by giving a place in your Journal to the few following observations, which I intend as partly explanatory of some statements in my Report (Br. and For. Med. Rev, No. XLIV), and partly as a reply to objections that have been made to some of these statements by your own correspondents and by others. My animadversions on the Homœopathic treatment would be much more effective if I could vouch for the accuracy of a statement recently made to me—which I myself believe, but cannot positively assert to be true—viz., that in the Wiesen district hospital of Vienna, a physician has been treating all his patients with *aqua colorata* only, and, on being accused of doing so, and therefore threatened with dismissal, has retained his situation by producing his books and showing better therapeutic statistics than his colleagues!

*Peritonitis.*—The startling results obtained by Fleischmann in peritonitis, caused me to make it the subject of strict inquiry, and I found that its idiopathic and too fatal form, so frequent here, was unknown, or at least very rare in Vienna. There it occurs as a subacute, tubercular inflammation usually of circumscribed extent. Such cases I have seen recover under the use of *extract. graminis,* and, although no cases occurred during my visits to the Homœopathic Hospital, I have no doubt that the infinitesimals would be equally efficacious. The disease is truly peritonitis, but of so peculiar character that there cannot be a more flagrant instance of the deceptive nature of the statistics of names, than to compare it with ours.

*Gout.* This disease, or, as it is called, "die gichtische Dyscrasie," as evidenced by symptoms of portal congestion, is exceedingly common, and exercises its modifying influence on many diseases. Its recognition in relation to those of the eye, forms, indeed, one of the peculiarities of the Vienna Oculistic School. The prevalent use of fermented liquors, wine, and beer, and greasy sauces, are looked upon as its causes. It is at best a hypothetical erratic gout, and comparison with ours leads but to misconception.

*Bronchitis.* This is a comparatively rare disease in Vienna, and, compared with the frequency of pneumonia, it is most strikingly so.

I could name some other peculiarities of the Vienna class of diseases; no less striking, and still less easily explicable. Thus, during the ten years preceding 1845, we have in Fleischmann's tables, as published in the Austrian Journal of Homœopathy, 4 inflammations of the aorta, all cured; 2 of inflammation of the spinal cord, cured; 3 of ulceration of the stomach, discharged unimproved, and 2 cured; 4 cases of white swelling of the knee-joint, cured.

*Intermittent fever.* Of the 30 patients who had intermittent fever and were treated by Fleischmann, the average number of attacks was 4·7; while under Skoda's treatment, the average number could not be more than 3, and was most probably under this. The number of his patients was, I believe, at least as great as Fleischmann's—if not greater; his cases were as severe; the patients were afflicted with the same epidemic, during the same season, inhabited the same town,—many of them the same quarter—and belonged to a similar rank in life. They were treated in undoubtedly less advantageous circumstances with respect to food and lodging—yet all were cured. In no one case was the number of attacks more than 4: and in none did the fever recur while they continued under observation: they were therefore cured speedily, safely, and effectually. If it be true that none but homœopathic remedies are capable of effecting this, then Skoda's remedies must have been homœopathic—but they were not so, were not infinitesimal.

Were homœopathy true, it would be very extraordinary that a physician practising empirically like Skoda, should have so simply and effectually cut short the disease, while a scientific homœopathist of experience, with his carefully and scientifically selected array of China, Quinin, Ipecacuanha, Nux vomica, Arsenic, and Aconite, was unable to do so. It is still more singular that, although it is evident from the results of Skoda's practice that Quinin was indicated in the greater number of cases of this epidemic, it fared no better with those of the homœopathic patients who got this remedy infinitesimally than with the others.

In corroboration of the obvious inference from these facts, we might quote the confessions of the homœopathists themselves, or of those who have been reconverted to allopathy. Thus, Kopp states that this disease can neither be certainly, quickly, nor pleasantly cured by the infinitesimals. Nay, he quotes avowed homœopaths, Gross, Rummel, Œgidi, and Hauptmann, to the same effect. It must be admitted, however, that while acknowledging this, and in certain cases flying to allopathic doses of quinine, these gentlemen look upon their non-success as merely an imperfection of the youth of their system, and hope yet to discover the true, i. e. homœopathic, specific for ague. Gross expressly states that ordinary homœopathic remedies only cured ague exceptionally, and that antipsoric remedies alone fulfilled his expectations. But, as Kopp says, this is too bad to submit the poor patients to a method of treatment which allows of the renewal of the dose only after 4 or 6 weeks.* Kopp also tells us as the result of his own experience (s. 184), that even where the remedy (China) was homœopathically indicated, and given in infinitesimal (Hahnemann'scher) doses, it had no influence over the appearance or diminution of the intermitting fever; while, on the contrary, when given in substance in sufficient dose (as sulphate of quinine, 2 grs.), it speedily cut the fever short. I think there can be, therefore, but one opinion as to which method of treatment for this disease is the preferable. But what are we to think of a system whose very foundation-stone is so unstable? For, as is well known, it was the action of cinchona that first led Hahnemann to think of his new theory.

*Dysentery.* In my Report under the head of dysentery, one case will be found in which the amelioration of the symptoms was curiously coincident with the administration of the Homœopathic remedies. I say coincident, because in the first case, which greatly resembled it, the same remedy was of no avail; and we also know that however medical men have been accustomed to regard the disease as demanding the most active treatment, more especially in warm climates, we know that many cases of dysentery may, nay do, recover without it. I may refer for an illustration of this fact to the case of a patient, a physician, as narrated by himself. Away from all friends and without medicine, he was seized with dysentery while voyaging on the Nile in an open boat; after a partial recovery he relapsed, and using nothing but hot fomentations finally recovered, after about a week's illness, and this during continued exposure. He adds, "during a precisely similar attack in India, I got 216 grs. calomel, was bled at the arm, had 50 leeches applied, and used mercurial frictions; in spite of this heroic treatment I recovered, but in such a state as to require 6-8 week's furlough to recruit."†

Again, Dr. Smith ‡ mentions the case of a Frenchman who cured himself of

* Denkwürdigkeiten in der ärztlichen praxis, s. 290.

† Cumming's Notes of a Wanderer in Search of Health, p. 277.

‡ On the Diseases of Peru, Edinb. Med. and Surg. Journal, vol. lvi.

a dysentery after 13 relapses, by semistarvation; and of a medical man who trusted to this alone for the cure of his patients, and found it undoubtedly successful in many cases, though in others it was too reducing. There can, therefore, be no doubt that dysentery *may* recover without any active treatment whatever. Skoda's treatment consists in giving injections of tincture of opium or sulphate of zinc, and, if these fail, of corrosive sublimate, which he looks upon as the *ne plus ultra*. In corroboration of the occasional usefulness of these stimulating injections, Dr. Smith (loc. cit.) mentions the case of a boy who, instead of a little rum and water as a drink, got a whole syringeful of pure pale rum per anum. The boy became intoxicated, fell asleep, and awoke quite recovered from a dysentery of some months' standing. In Skoda's hands these injections prove very successful.

*Pneumonia.* In the cases of pneumonia observed by me in the Homœopathic Hospital, there were one or two coincident cures; in most, however, the symptoms were either aggravated or run their course, or merged in others, apparently uninfluenced by the remedies. I question not the homœopathic correctness of the selection of the remedies, for most of the cases were treated with *Phosphor.*, and Fleischmann has said, that any pneumonia incurable by Phosphor., is as yet incurable homœopathically, and other homœopaths have corroborated this. Who am I, therefore—heretic, as I avow myself—that I should gainsay this deliberate opinion of the initiated, founded on the experience of years? In regard to the aggravations, I acquit the remedies of all blame in their production, just as I deny them all merit in the ameliorations and eventual cure. Surely it is much more natural and consonant with reason to believe that these and all the other cures hitherto of the homœopathists are natural cures—effects of the too-much neglected *vis medicatrix*. And I will even go so far as to doubt whether Fleischmann himself will not here agree with me; for I have heard him say that, out of 1000 cases treated, he did not believe that above 200 were *cured;* the rest got well. And where is the proof that even these 200 did not also get well? Were not even the most remarkable cures merely coincidences? Every quack medicine, however ridiculous or inert, either in an allopathic or homœopathic point of view, has also, as everybody knows, its numerous extraordinary cures, or coincidences. It would be remarkable if homœopathy had none. Even under Skoda's treatment such occur; yet, according to our ideas, his treatment is equivalent to nothing; according to the homœopathists, it must be worse than nothing. And yet, as we have seen, the patients recovered perfectly, aye, and speedily,—the average duration of treatment in acute cases being under a fortnight, about 9 to 10 days. A few cases of a chronic character remained in the state of hepatization for weeks, but these had been generally some time ill previous to admission. The most remarkable fact with respect to the pneumonic patients treated without active interference is, that however much they may suffer for a time, on the disease breaking, the recovery is extremely rapid, insomuch that the general health seems almost as strong in a day or two as it was before the attack: there is no tedious convalescence. This remark applies both to those treated by Skoda, and by the homœopathists. The completeness of the recovery was ascertained in one by dissection. The patient, a woman, died a week or two after dismissal from a heart complaint under which she had long laboured. Both of her lungs were found perfectly healthy, and permeable by air in every direction, although one had been completely hepatized.

Skoda does not, however, always trust to Nature. When the dyspnœa is very great at the commencement of pneumonia, or pleurisy, he bleeds, but never after hepatization or effusion has taken place; as he believes that abstraction of blood lessens the chance of a speedy and complete absorption of the exudation, which of itself will relieve this symptom. If the cyanosis be accompanied by a secretion of tough mucus, an emetic is given.

In reference to the relative mortality and cure of pneumonia in different situations, and under various methods, the following rough approximations, taken from data most at hand, will be admitted to be of some importance:

The admissions and deaths from pneumonia in 1842, in the undermentioned hospitals, were as follows: Edinburgh, 42 a. 16 d.; Aberdeen, 10 a. 3d.; Dumfries, 10 a. 2 d.; Glasgow, 33 a. 9 d.; Dundee, 27 a. 4 d.; Inverness, 2 a. 0 d; Perth, 1 a. 1 d.; total 125 admitted and 35 dead—28 per cent., or about 1 in 3·54.* This is a very large proportion of deaths, but a certain part, perhaps a large part, of this is due to other causes than the treatment, such as the age of the patients, their previous privations and intemperate life, and, it may be, the existence of chronic disease.

From the statistical reports on the health of the army, it appears that the admissions and deaths from pneumonia in Britain and our temperate colonies, have been as follows: United Kingdom, 657 a. 37 d., or 1 in 18; Gibraltar, 2515 a. 56 d., or 1 in 45; Malta, 1370 a. 44 d., or 1 in 31; Ionian Islands, 2186 a. 81 d., or 1 in 27; Nova Scotia and New Brunswick, 1505 a. 56 d., or 1 in 27; Canada, 2774 a. 99 d., or 1 in 28; Cape of Good Hope, 673 a. 22 d., or 1 in 31. The average age of the men may be taken at from 25 to 30. Their condition is, of course, superior to the ordinary run of our hospital patients, though perhaps their diathesis is therefore all the more "phlogistic," and, though not free from the effect of intemperance, they must be generally free from chronic complications. Their station and manner of life is not superior to that of Fleischmann's patients, yet his extended statistics are inferior to any of the above. During 10 years he treated 347 pneumonic patients. with 20 deaths—5·70 per cent., or 1 in 17·35., and this, it will be understood, among patients of a younger class than in most of the other cases. Where, then, is the boasted superiority of homœopathic treatment? Again, if we take Skoda's average of the last three years, viz. 392 admissions and 54 deaths, i. e. 13·7 per cent., or 1 in 7·26, to be a sample of what can be done with ordinary unpicked hospital patients, by a little less active interference and trusting more to Nature, and find that we have a reduction of the mortality to one half, may we not expect the results to be still more astonishingly favorable among patients in a manner picked with respect to age and constitution, I demand once more, and in a bolder tone, where is the boasted superiority of homœopathic treatment?

I will not occupy your space with any extended observations in defence of the general inferences in my Report, as they have been attacked by the homœopathists. It is hopeless to make any impression on them. Before concluding, I must, however, say a few words on the present aspect of homœopathy, as confirmed by the opinions and practice of some of its own advocates. There is, indeed, confusion and war in the very camp. The old original doctrine of Hahnemann is virtually abandoned by all. The new theories are legion. The holders of the one theory are ruthlessly falling foul of those of the others, the ultra-infinitesimalists are warring with the more rational dose-givers; the "spezifikers" are rejected by "those who have more deeply studied the subject."†

The only thing they seem agreed upon, is the necessity of *proving* the medicines and choosing their remedies according to the principle of similitude. But they who will be at the trouble to examine the records of these provings, will see how much absurdity is mingled with a little sense, and will also perceive how difficult in most cases must have been the choice of a remedy had not the

* Vide a paper by Mr. Thomson, Edinb. Med. and Surg. Journal, No. 158.

† Vide Madden, Homœop. and Med. Ref., &c. &c.

despised allopathy supplied them with her experience. And they who will take courage—it does not require much—and *prove* the medicines on themselves, as I have done, will see how little is to be dreaded from infinitesimal doses; and how little is to be trusted to a system which substitutes mere nervous feelings constantly occurring without the use of drugs, for manifestations of the physiological action of those drugs.

I consider that the extension of homœopathy to the veterinary branch of medicine furnishes a very excellent proof of its inutility, in so far as the indications for the use of the remedies are taken from the results of provings on man, and many of these, particularly vegetable ones, have actions so different in kind and degree on animals from what they have on man; not but that there, as in human medicine, it will prove strikingly useful, because there, even more than in human medicine, the heroic system is carried too far. A little more attention to the events of the stable might suffice to convince the homœopathists of the utility of active treatment when skilfully and judiciously employed, as well as of its injurious influence when the contrary is the case. Here, at least, the influence of imagination is wanting; the action of the drugs, remedial or not, is developed simply, and uncomplicated by mental influence.

To conclude, while I believe that physicians have, to a certain extent, been deceiving themselves for years—centuries shall I say—as to the efficacy of many drugs, have been masking symptoms by heroic treatment, and often falsely believing they were thereby curing diseases, I believe, at the same time, that homœopathists are now deceiving themselves infinitely more—are allowing Nature to kill or cure as she pleases—are walking up to the first part of Chomel's golden axiom, that "the first law of therapeutics is not to do harm," and turning their backs on the equally important second law, which commands them "to do good," and which, so long as they remain homœopathists, in the present, or even in any sense of the word, they are for ever shut out from doing.

I remain, my dear Sir, yours truly,

GEORGE W. BALFOUR.

P.S. The following errata have crept into my last letter:

Page 567, line 2d from bottom, *between* practicable *and* as, *insert* certainly requisite.

Page 577, line 22d from bottom, *instead of* for three weeks he had a regular fever, *read* three weeks previously he had had one or two attacks of regular fever.

Page 584, line 25th from top, *before* elsewhere, *insert* over dull spaces—respiration clear—bronchial.

